# Flavopiridol Mitigates the Progression of Monocrotaline-Induced Pulmonary Hypertension in Rats by Targeting Cyclin-Dependent Kinase 9

**DOI:** 10.1007/s10557-021-07285-w

**Published:** 2022-01-28

**Authors:** Qi Jia, Zhiqiang Hu, Nannan Song, Weike Mao

**Affiliations:** Department of Anesthesiology, Union Hospital, Tongji Medical College, Huazhong University of Science and Technology, Wuhan, China

**Keywords:** Pulmonary hypertension, Cyclin-dependent kinase 9 (CDK9), Flavopiridol, Proliferation, Apoptosis

## Abstract

**Purpose:**

To investigate the role of cyclin-dependent kinase 9 (CDK9) and the therapeutic potential of a CDK9 inhibitor (flavopiridol) in monocrotaline (MCT)-induced pulmonary hypertension (PH).

**Methods:**

For the in vivo experiments, rats with PH were established by a single intraperitoneal injection of MCT (60 mg/kg). After 2 weeks of MCT injection, rats were then treated with flavopiridol (5 mg/kg, i.p., twice a week) or vehicle for 2 weeks. For the in vitro experiments, human pulmonary artery smooth muscle cells (HPASMCs) were treated with flavopiridol (0.025-1 μM) or vehicle under hypoxic conditions. Hemodynamic recording, right ventricle histology, lung histology, and pulmonary arterial tissue isolation were performed. The expression levels of CDK9, RNA polymerase II, c-Myc, Mcl-1, and survivin were determined by qRT-PCR and western blotting, and the proliferation and apoptosis of rat pulmonary arterial tissues and/or HPASMCs were also assayed.

**Results:**

Compared to the control group, CDK9 was upregulated in pulmonary arterial tissues from MCT-induced PH rats and hypoxic cultured HPASMCs. Upregulation of CDK9 was associated with enhanced phosphorylation of the C-terminal domain (CTD) of RNA polymerase II (RNA pol II) at serine-2 (Ser-2), promoting the expression of prosurvival and antiapoptotic proteins (c-Myc, Mcl-1, and survivin). Furthermore, treatment with flavopiridol (5 mg/kg) significantly alleviated pulmonary artery remodeling and partially reversed the progression of MCT-induced PH. Consistently, flavopiridol (0.5 μM) treatment decreased the proliferation and induced the apoptosis of cultured HPASMCs under hypoxic conditions. As a result of CDK9 inhibition and subsequent inhibition of RNA pol II CTD phosphorylation at Ser-2, flavopiridol decreased c-Myc, Mcl-1, and survivin expression in isolated pulmonary small arteries, leading to cell growth inhibition and apoptosis.

**Conclusion:**

Flavopiridol mitigates the progression of MCT-induced PH in rats by targeting CDK9.

## Introduction

Pulmonary hypertension (PH) is a devastating cardiopulmonary disorder with poor prognosis and limited curative options. Despite receiving treatments, approximately 10–15% of patients with PH die within 1 year of medical follow-up [1–3]. The remodeling of small pulmonary arteries is an important pathological characteristic of PH [3, 4]. Recently, a novel cancer-like concept for PH has emerged because some similarities between cancer and PH have been confirmed [5–7]. Similar to the hallmarks of cancer cells, pulmonary artery smooth muscle cells (PASMCs) are characterized by overproliferation and resistance to apoptosis [8, 9]. However, the underlying mechanisms of pulmonary vascular remodeling are not fully understood.

Cyclin-dependent kinases (CDKs) can be divided into partially overlapping classes of cell cycle regulators (e.g., CDK1, 2, 4, 6, and 7) and transcriptional regulators (e.g., CDK7, 8, 9, and 10–13) [10]. CDK9 is a key catalytic subunit of positive transcription elongation factor b (P-TEFb) that promotes transcription elongation by phosphorylating the C-terminal domain (CTD) of RNA polymerase II (RNA pol II) at Ser-2 [11]. Activation of CDK9 enhances the expression of several signal-responsive proteins that regulate proliferation and apoptosis, such as a prosurvival protein (c-Myc) and antiapoptotic proteins (Mcl-1 and survivin) [12, 13]. The dysregulation of CDK9 activity or expression has been shown to be associated with several diseases, including cancer, cardiac hypertrophy, and acquired immunodeficiency syndrome (AIDS) [11, 14, 15]. Compared to normal tissues, CDK9 overexpression and/or hyperactivity has been observed in several types of cancers (e.g., melanoma) [14, 16–18]. The upregulation of several prosurvival and antiapoptotic proteins (e.g., c-Myc, Mcl-1, and survivin) in cancer cells has been confirmed to contribute to their overproliferation and resistance to apoptosis [19, 20]. Intriguingly, enhanced c-Myc, Mcl-1, and survivin expression has also been observed in both PH patients and pulmonary artery smooth muscle cells (PASMCs) in PH models [21, 22]. However, whether the enhanced proliferation and resistance to apoptosis of PASMCs in PH is due to the CDK9-mediated overexpression of these targeted genes remains unknown.

Additionally, the inhibition of CDK9 by selective blockers or shRNAs leads to a block in transcription elongation and the suppression of short-living antiapoptotic proteins (e.g., Mcl-1), resulting in an increased rate of apoptosis and antitumor effects [13, 18, 20]. Flavopiridol is one of the most studied CDK9 inhibitors and has been tested in antitumor and cardiac hypertrophy clinical trials [23–25]. Flavopiridol displays potent inhibition against CDK9, and its mechanism of action involves the inhibition of CDK9-mediated transcription elongation [24]. Flavopiridol is known to bind tightly to the ATP binding site of CDK9 in a noncompetitive manner to inhibit the CDK9-mediated phosphorylation and activation of RNA pol II, thereby affecting RNA pol II-dependent transcription. Mcl-1, Bcl-2, and Bcl-xL have been shown to be inhibited by flavopiridol treatment, and it is possible that these events are mediated through CDK9 inhibition [26, 27].

Several proteins, including hexamethylene-bisacetamide-induced protein 1/2 (HEXIM1/2), La-related protein 7 (LARP7), and methylphosphate capping enzyme (MePCE) have been shown to negatively regulate the kinase activities of CDK9 or P-TEFb and have also been reported to be involved in CDK9-related diseases [28, 29]. For example, LARP7 downregulation has been confirmed in several cancers, which subsequently potentiates CDK9-mediated transcription elongation and promotes cancer growth and metastasis [30]. However, it remains unknown whether these regulatory proteins are involved in the pathological process of PH.

In the present study, we investigated the roles and underlying mechanism of CDK9 in the cancer-like phenotype (overproliferation and resistance to apoptosis) of PASMCs in PH rats and we sought to elucidate whether a CDK9 inhibitor (flavopiridol) influences the pathogenic progress of PH by targeting CDK9.

## Materials and Methods

### Experimental animals and pulmonary artery hypertension models

Adult young male Sprague Dawley rats (200–250 g, 2–3 months old) were purchased from the Experimental Animal Center of Weitonglihua Co., Ltd. (Beijing, China). Monocrotaline (MCT) was purchased from Sigma-Aldrich (St. Louis, MO, USA), and it was dissolved in 1.0 N HCl at a concentration of 40 mg/ml, adjusted to pH 7.4 with 1.0 N NaOH and diluted with distilled water. Flavopiridol was purchased from Selleckchem (Burlington, ON, Canada) and dissolved in dimethyl sulfoxide (DMSO, Sigma-Aldrich). The rats were intraperitoneally injected with MCT (60 mg/kg) or vehicle (normal saline), and once PH was established (2 weeks after MCT injection), the rats with PH were randomly divided into two groups and treated with either flavopiridol (5 mg/kg, i.p., twice a week) or vehicle (0.01% DMSO) for 2 weeks. All experiments involving animals were performed with the approval of the Animals Care and Use Committee of Huazhong University of Science and Technology (Wuhan, Hubei, China). Up to three rats were housed per cage, and the animals were provided free access to water and food with constant room temperature and humidity maintenance under a 12–12 h light–dark cycle.

### Assessment of right ventricle function and hypertrophy

The right ventricle systolic pressure (RVSP) of rats was recorded as previously reported [31]. Briefly, at the end of the experiment (4 weeks after MCT injection), the rats from each group were anesthetized (pentobarbital, 50 mg/kg, i.p.) and intubated. The diaphragm was then surgically exposed through the abdomen, and a 25-gauge needle connected to a pressure transducer (AD Instrument, Sydney, NSW, Australia) was inserted into the right ventricle (RV) through the diaphragm. The RVSP was continuously recorded for 10–15 min using a PowerLab data acquisition system (AD Instruments). Following hemodynamic recording, the heart and lungs were removed en bloc. The right ventricle (RV) and left ventricle plus interventricular septum (LV + S) were then separated and weighed to calculate the RV hypertrophy index (RV/[LV + S]).

### Histology and morphometric analysis

The collected lungs were perfused with normal saline and fixed in 4% paraformaldehyde overnight. The representative cross-sections of the lungs that included the peripheral and the central pulmonary arteries were then sampled and embedded in paraffin blocks. Serial Sects. (5 μm thick) were then prepared and stained with hematoxylin and eosin to assess vascular pathology. The percent wall thickness (WT %) of arteries (15–150 μm) was calculated using the following formula as previously described: WT % = 2 × WT/external diameter (ED) × 100. The percent wall area of arteries was also measured.

### Preparation of pulmonary arterial tissue

The PH and control rats were anesthetized with pentobarbital sodium (200 mg/kg, i.p.), and the hearts and lungs were removed after a midline thoracotomy. Intrapulmonary arteries from the lung lobes were aseptically excised and placed in PBS solution. The adherent fat and connective tissues were then carefully removed under a dissecting microscope. The adventitia was scraped using forceps, and the endothelium was carefully removed. The pulmonary arterial tissues were immediately flash-frozen in liquid nitrogen and stored at − 80 °C for use in the subsequent experiments. Notably, pulmonary arterial tissues from the control group (0 week) and MCT groups (1, 2, 3, and 4 weeks after MCT injection) were used to evaluate the effects of MCT on CDK9 mRNA and protein expression.

### Cell culture and in vitro experiments

Human pulmonary artery smooth muscle cells (HPASMCs, passages 4–6, Lonza, USA) were cultured in DMEM at 37 °C under humidified 5% CO_2_. For in vitro hypoxic experiments, HPASMCs were cultured in an incubator equilibrated with 3% O_2_ (in N_2_), while control cells were cultured in an incubator equilibrated with room air (21% O_2_). Flavopiridol (0.025–1 μM in vehicle) or an equal volume of vehicle (0.01% DMSO) was added to DMEM during the final 24 h of exposure to normoxic or hypoxic conditions.

### Protein preparation and Western blotting

HPASMCs were lysed in RIPA buffer (Thermo Scientific, Rockford, IL) supplemented with a protease inhibitor cocktail (Roche, Mannheim, Germany). Cell lysates were centrifuged at 12,000 rpm for 15 min at 4 °C, and the supernatants were then used as protein samples. Equal amounts of protein (30 μg) from each cell type were separated on SDS–polyacrylamide gels and transferred to nitrocellulose membranes (Bio-Rad, Munich, Germany). After blocking with 5% skim milk in Tris-buffered saline supplemented with 0.1% Tween 20 for 1 h at room temperature, the membranes were incubated at 4 °C overnight with the following antibodies: anti-CDK9 (CST, 1:500), anti-RNA pol II (Abcam, 1:1000), anti-phospho-RNA pol II (Ser-2) (Abcam, 1:1000), anti-Mcl-1 (Abcam, 1:1000), anti-c-Myc (Abcam, 1:1000), anti-survivin (CST, 1:500), and anti-β-actin (Abcam, 1:3000). The protein levels were normalized to β-actin. The gel bands were visualized with Amersham ECL prime Western blotting detection reagent (GE Healthcare, Little Chalfont, UK), and the band density was quantified with ImageJ software (National Institutes of Health, Bethesda, MD).

### Real-time PCR

Total RNA from rat pulmonary arteries and HPASMCs was isolated using TRIzol and reverse transcribed with gene-specific primers. Small pulmonary arteries (< 1000 μm) from PH or control rats were used for RNA extraction. Real-time PCR was performed as previously described [3]. Specific primers are listed in Table 1.

**Table 1:** Primer sequences of genes

Genes	Primer sequences(5’-3’)
Human-CDK9	Sense:ATGGCAAAGCAGTACGACTCG Antisense:GCAAGGCTGTAATGGGGAAC
Human-GAPDH	Sense:5’-GAAGGTGAAGGTCGGAGT-3’ Antisense:5’-GAAGATGGTGATGGGATTTC-3’
Rat-CDK9	Sense:5’-CCTCCGGCACTCGTTGGCTG-3’ Antisense:5’-GATTTCGGCTCTGGTTGGT-3’
Rat-c-Myc	Sense:5’-CAACGTCTTGGAACGTCAGA-3’ Antisense:5’-TCATCTGCTTGAACGGACAG-3’
Rat-Mcl-1	Sense:5’-TCATCTCCCGCTACCTGC-3’ Antisense:5’-ACTCCACAAACCCATCCC-3’
Rat-survivin	Sense:5’-ACCACCGGATCTACACCTTCAAGA-3’ Antisense:5’-ATTCTCGGTAGGGCAGTGGATGAA-3’
Rat-Larp7	Sense:5’-ACGGTATATGTGGAGTTGC-3’ Antisense:5’-AACAACATTGCCACATTTCC-3’
Rat-HEXIM1	Sense:5’-CAGAATTGAGCTGCTTGGA-3’ Antisense:5’-CCAGTGGATAAGTCCTCCC-3’
Rat-HEXIM2	Sense:5’-AGACGAAGACTTCTGGTGG-3’ Antisense:5’-AATCTCCCTTCCTGGTTGC-3’
Rat-MePCE	Sense:5’-TCGTCACGGGTAACTATGTC-3’ Antisense:5’-CGGAACATTCTCTTCAGCC-3’
Rat-GAPDH	Sense:5’-GCCCATCACCATCTTCCAGGAG-3’ Antisense:5’-GAAGGGGCGGAGATGATGAC-3’

### Cell counting kit-8 (CCK8)

HPASMCs were seeded in a 96-well plate at a final density of 3 × 10^3^ cells/well [9] and incubated in growth media supplemented with flavopiridol (0.025–1 μM) for 24 h. Subsequently, the medium was replaced with fresh medium containing 10 μl of 2-(2-methoxy-4-nitrophenyl)-3-(4-nitropheny)-5-(2, 4-disulfophenyl)-2H-tetrazole monosodium salt (CCK8, Dojindo, Japan) solution per 100 μl of medium. After 2 h of incubation at 37 ℃, the plates were read at 450 nm using a microplate reader (Thermo, USA). Ten wells were used for each treatment.

### 5-Ethynyl-2′-deoxyuridine (EDU)

EDU (RIBOBIO, China) is a thymidine analog that is incorporated into replicating DNA in place of thymine during cell proliferation. The specific activity of the cellular DNA in response to the Apollo fluorescent probe dye is an indicator of cell proliferation. Cells were seeded in a 96-well plate at a density of 3 × 10^3^ cells/well [9], and each group was assayed in triplicate. After 12 h of synchronization, the cells in each group were treated for 24 h according to the experimental design, and the medium was replaced with medium containing 25 μM EDU as well as the stimulating factor and inhibitor. Subsequently, after incubation for 18 h, the cells were stained, imaged, and counted as the mean of five randomly selected fields under 400 × magnification.

### Statistical analysis

All experiments were performed in triplicate independent experiments. The results are presented as the mean ± S.E.M., and SPSS version 16.0 (IBM, New York, NY, USA) was used for all the statistical analyses, except where noted. Student’s *t*-test and one-way ANOVA with Tukey–Kramer multiple comparisons were used to assess the significance of differences between two or among multiple groups, respectively. Statistical significance was defined at *P* < 0.05 for all tests.

## Results

### CDK9 is Overexpressed in PH

**Fig.1:**
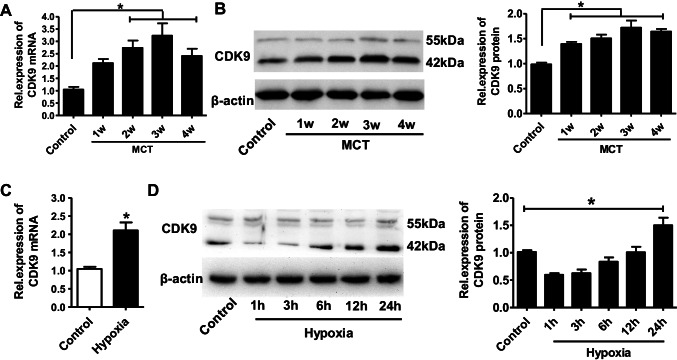
CDK9 is upregulated in pulmonary hypertension in vivo and in vitro. **(A)** Relative CDK9 mRNA expression in pulmonary arterial tissues of rats during development of MCT-induced PH (1- 4 weeks). (**B)** Respective images and the summarized data of CDK9 protein expression in pulmonary arterial tissues of rats during development of MCT-induced PH. **(C)** CDK9 mRNA expression in HPASMCs under control and hypoxia conditions. **(D)** Respective images and the summarized data of CDK9 protein expression in HPASMCs under hypoxia for 1–24 h. MCT, monocrotaline. Statistical analysis was performed with one-way ANOVA followed by Tukey–Kramer Multiple Comparison or an unpaired the student’s *t-*test, *P < 0.05, **P < 0.01. All values are expressed as the mean ± SEM, n = 5 in each group

First, to investigate whether CDK9 is involved in the development of PH, we measured CDK9 expression in pulmonary arterial tissues from rats with MCT-induced PH. As shown in Fig. 1A, CDK9 mRNA expression was significantly increased at 2, 3, and 4 weeks after MCT exposure (~ 2.7, 3.2, and 2.4-fold, respectively) compared to that of the control group. Consistent with the upregulation of CDK9 mRNA expression, CDK9 protein expression was also significantly upregulated at 1, 2, 3, and 4 weeks after MCT exposure (~ 1.4, 1.5, 1.7, and 1.5-fold, respectively) compared to the control group. Similarly, in an in vitro experiment, CDK9 mRNA expression was also markedly increased in HPASMCs exposed to hypoxia for 24 h hypoxia exposure (~ 2.1-fold, Fig. 1C), compared to the normoxic HPASMCs. Interestingly, CDK9 protein expression in the hypoxia group rapidly decreased upon the hypoxia treatment (1–3 h), but it was followed by a gradual increase over time and was ~ 1.5-fold greater than that observed in the control group at 24 h (Fig. 1D).

### Flavopiridol Mitigates the Progression of MCT-induced PH in Rats

**Fig. 2:**
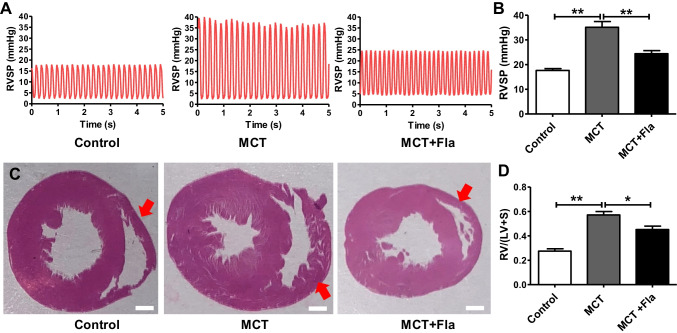
Flavopiridol mitigated the progression of MCT-induced pulmonary hypertension in vivo. **(A-B)** Representative curves **(A)** and the summarized data **(B)** of right ventricle systolic pressure (RVSP) in the control, MCT and MCT plus flavopiridol groups. **(C)** Representative examples of hypertrophied heart in the control, MCT and MCT plus flavopiridol groups. Red arrows indicated the hypertrophied right ventricle. Scale bar, 2 mm. **(D)** Summarized data of RV hypertrophy index (RV/[LV + S]) in the control, MCT and MCT plus flavopiridol groups. MCT, monocrotaline; Fla, flavopiridol; RV, right ventricle; LV, right ventricle; S, septum. Statistical analysis was performed with one-way ANOVA followed by Tukey–Kramer Multiple Comparison. *P < 0.05, **P < 0.01. All values are expressed as the mean ± SEM. n = 6 in each group

To further investigate the functional role of CDK9 in PH, we assessed whether CDK9 inhibition by flavopiridol treatment mitigates the progression of MCT-induced PH in rats. As expected, the baseline RVSP in the MCT group (35.1 ± 2.3 mmHg) was significantly higher than that in the control group (17.7 ± 0.7 mmHg), and CDK9 inhibitor flavopiridiol (5 mg/kg) treatment significantly decreased the RVSP (24.4 ± 1.3 mmHg, *P* < 0.05) of MCT-treated rats when compared to the untreated MCT rats (35.1 ± 2.3 mmHg) (Fig. 2A, B). Additionally, as shown in Fig. 2C-D, MCT promoted a significant increase in the RV hypertrophy index (0.57 ± 0.01, *P* < 0.05) compared to that observed in the control group (0.27 ± 0.01), while flavopiridol mitigated PH as the RV hypertrophy index was significantly lower in the flavopiridol treated MCT group (0.45 ± 0.01, *P* < 0.05) than in the untreated MCT group (0.57 ± 0.01).

### Flavopiridol Attenuates the Vascular Remodeling of MCT-induced PH in Rats

**Fig. 3:**
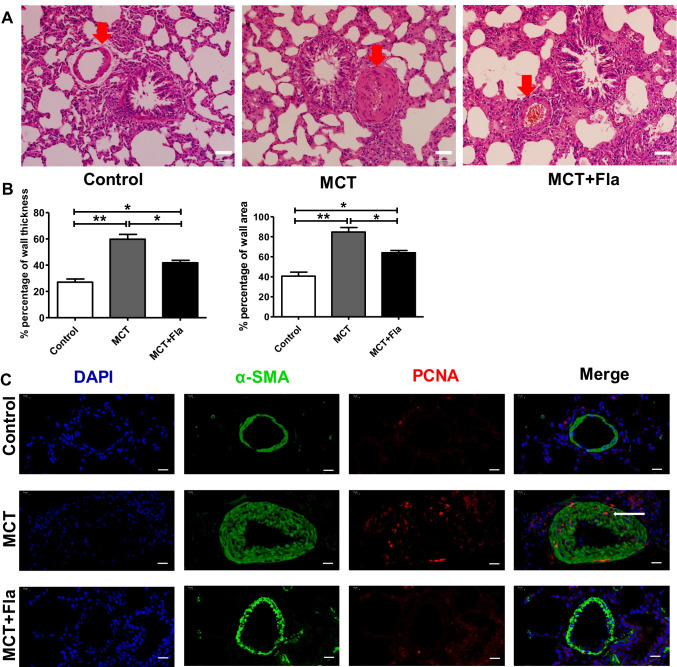
Flavopiridol attenuated the vascular remodeling of MCT-induced pulmonary hypertension in rats. **(A)** Representative photograph of lung sections from the control, MCT and MCT plus flavopiridol groups. Red arrows indicated the small pulmonary arteries. Scale bar, 50 μm. **(B)** Summarized data of wall thickness and wall area of small pulmonary arteries from the control, MCT and MCT plus flavopiridol groups. **(C)** Confocal images of α-SMA and PCNA positive cells in small pulmonary arteries from the control, MCT and MCT plus flavopiridol groups. White arrow indicated PCNA positive smooth muscle cells. Scale bar, 20 µm. MCT, monocrotaline; Fla, flavopiridol: Statistical analysis was performed with one-way ANOVA followed by Tukey–Kramer Multiple Comparison. *P < 0.05, **P < 0.01. All values are expressed as the mean ± SEM. n = 5 in each group

Because the remodeling of small pulmonary arteries is an important pathological characteristic of PH [2, 8, 9], we next assessed whether a reduction in pulmonary vascular remodeling of small pulmonary arteries accounts for improved hemodynamics in flavopiridol-treated rats. As shown in Fig. 3A, B, PH in MCT-treated rats was associated with an increase in the media wall thickness of pulmonary arteries, which was significantly attenuated by flavopiridol treatment. Furthermore, as shown in Fig. 3C, immunofluorescence analysis of lung tissue indicated that compared to the control group, the expression of proliferating cell nuclear antigen (PCNA, marker of cell proliferation) in the pulmonary arteries was significantly increased in the MCT-treated rats and decreased when these rats were administered a CDK9 inhibitor (flavopiridol).

### Flavopiridol Inhibits the Overproliferation and Promotes the Apoptosis of HPASMCs under Hypoxia in vitro

**Fig. 4:**
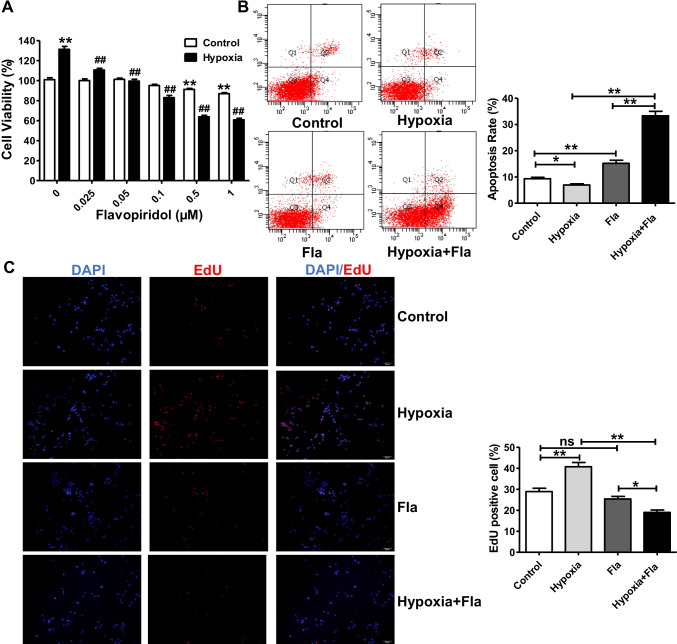
Flavopiridol inhibited the overproliferation and promoted the apoptosis of HPASMCs following subjected to hypoxia in vitro. **(A)** Effect of flavopiridol on the cell viability of human pulmonary artery smooth muscle cells (HPASMCs) in the control and hypoxia groups (n = 10). * vs control, ^**#**^ vs Hypoxia. **(B)** Effects of hypoxia and flavopiridol on the apoptosis rate of HPASMCs (n = 6). **(C)** Representative images (left panels) and the summarized data (right panels) of EdU positive cells in the control, hypoxia, flavopiridol and hypoxia plus flavopiridol groups (n = 6). Fla, flavopiridol: Statistical analysis was performed with two way ANOVA or one-way ANOVA followed by Tukey–Kramer Multiple Comparison. *P < 0.05, **P < 0.01. All values are expressed as the mean ± SEM

The uncontrolled proliferation and resistance to apoptosis of PASMCs are the predominant factors of pulmonary vascular remodeling [2, 9]. Hypoxia is an important stimulus for HPASMC proliferation and apoptotic resistance. Therefore, the effects of flavopiridol on the proliferation and apoptosis of hypoxia-treated and control HPASMCs were investigated. As shown in Fig. 4A, the viability of HPASMCs in the hypoxia group (3% O_2_, 131.5 ± 2.9%) was significantly higher than that of HPASMCs in the control group (21% O_2_, 100.2 ± 1.6%). Furthermore, flavopiridol strongly inhibited HPASMC proliferation in a concentration-dependent manner in the hypoxia group, especially when flavopiridol was used at 0.5 μM, which caused a ~ 50% decrease in cell viability, whereas flavopiridol minimally influenced HPASMC cell growth in the control group. Thus, flavopiridol treatment reversed the hypoxia-induced overproliferation of HPASMCs.

Additionally, in agreement with the CCK-8 assay results, the number of apoptotic HPASMCs was remarkably decreased under hypoxic conditions (7.0 ± 0.4%) compared to that observed in the normoxic control (9.4 ± 0.5%), while flavopiridol (0.5 μM) significantly promoted the apoptosis of hypoxia-treated HPASMCs (from 7.0 ± 0.4% to 33.3 ± 1.7%, ~ fivefold) but only minimally increased apoptosis of untreated HPASMCs (from 9.4 ± 0.5% to 15.2 ± 1.2%, ~ 1.6-fold, Fig. 4B). Furthermore, hypoxia significantly increased the percentage of EdU-positive cells (40.8 ± 2.0%) compared to that observed in the normoxic control group (28.9 ± 2.0%), and flavopiridol dramatically reduced hypoxia-induced HPASMC overproliferation (from 40.8 ± 2.0% to 19.0 ± 1.1%, Fig. 4C).

### Flavopiridol Inhibits CDK9-mediated Transcription Elongation and Suppresses the Expression of Downstream Prosurvival and Antiapoptotic Proteins in Pulmonary Arterial Tissues in PH Rats

**Fig. 5:**
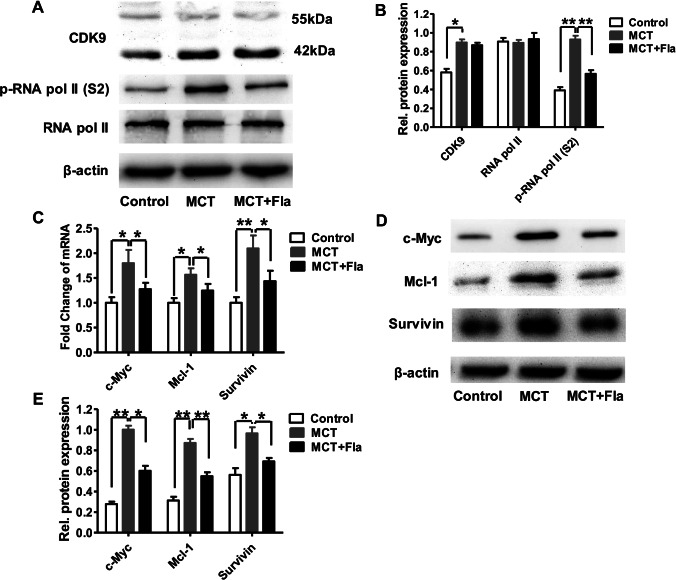
Flavopiridol inhibited CDK9-mediated transcription elongation and suppressed the expression of downstream target proteins of pulmonary arterial tissues in MCT-induced pulmonary hypertension. **(A-B)** Representative images **(A)** and the summarized data **(B)** of expressions of CDK9, RNA polymerase II phosphoforms at Ser-2 and RNA polymerase II in the control, MCT and MCT plus flavopiridol groups. **(C-E)** The mRNA **(C)** and protein **(D, E)** levels of expression of c-Myc, Mcl-1, survivin in the control, MCT and MCT plus flavopiridol groups. Statistical analysis was performed with one-way ANOVA followed by Tukey–Kramer Multiple Comparison. *P < 0.05, **P < 0.01. All values are expressed as the mean ± SEM. n = 5 in each group

As flavopiridol has been previously reported to be a potent CDK9 inhibitor [23], we further examined whether flavopiridol inhibits CDK9 expression or kinase activity. Compared to that observed in the control group, CDK9 expression was significantly upregulated in the MCT group, and flavopiridol had no effect on MCT-induced CDK9 expression (Fig. 5). Subsequently, we examined the effect of flavopiridol on CDK9 kinase activity by measuring the cellular levels of RNA polymerase II phosphoforms at Ser-2, which is the specific site for RNA polymerase II phosphorylation by CDK9. Figures 5A, B show that significantly higher phosphorylation of RNA polymerase II at Ser-2 occurred in the MCT group (0.93 ± 0.04, *P* < 0.05) than in the control group (0.39 ± 0.03). Interestingly, flavopiridol significantly reduced the phosphorylation of RNA polymerase II at Ser-2 in isolated pulmonary arterial tissues (from 0.93 ± 0.04 to 0.56 ± 0.04), but it had no effect on the total protein levels of RNA polymerase II in isolated pulmonary arterial tissues.

CDK9-mediated transcription of several prosurvival and antiapoptotic proteins (e.g., c-Myc, Mcl-1 and survivin) plays an important role in the proliferation and apoptosis resistance of cancer cells [19, 20], and the aforementioned prosurvival and antiapoptotic proteins are also involved in the pathology of PH [21, 22]. Thus, the effects of flavopiridol on the expression of c-Myc, Mcl-1, and survivin were examined. As shown in Fig. 5C-E, the mRNA and protein expression levels of c-Myc, Mcl-1, and survivin in isolated pulmonary arterial tissues of MCT-treated rats were significantly upregulated compared to those of the control group, and these downstream prosurvival and antiapoptotic proteins were decreased by flavopiridol treatment.

### CDK9-related Negative Regulators are Downregulated in PH

**Fig. 6:**
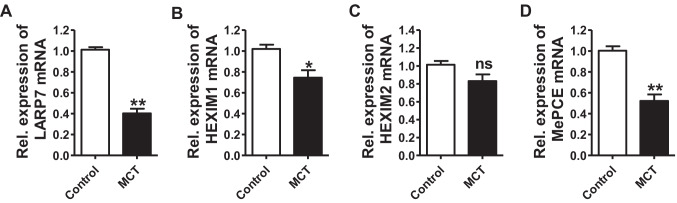
CDK9-related negative regulators are downregulated in pulmonary hypertension. **(A-D)** The mRNA expression of LARP7, HEXIM1/2 and MePCE in pulmonary arterial tissues isolated from the control and MCT-treated rats (n = 5 in each group). Statistical analysis was performed with the student’s *t-*test. *P < 0.05, **P < 0.01. All values are expressed as the mean ± SEM

Emerging evidence suggests that some CDK9-related negative regulators (e.g., HEXIM 1/2, LARP7, and MePCE) are also involved in CDK9-related diseases, e.g., cancers [28, 30]. Next, we examined whether these negative regulators are also involved in PH. Compared to the control group, decreased LARP7, HEXIM 1, and MePCE mRNA expression (~ 0.4, 0.7, 0.5-fold change) was detected in pulmonary arterial tissues isolated from MCT-exposed rats, while no significant difference was observed in HEXIM 2 mRNA expression between the control and MCT-treated rats (Fig. 6).

## Discussion

In the present study, we first revealed that CDK9 was upregulated in isolated pulmonary arterial tissues from MCT-induced PH rats in vivo and in hypoxic cultured HPASMCs in vitro*.* This upregulation of CDK9 was associated with increased CDK9-mediated phosphorylation of the CTD of RNA pol II at Ser-2. Second, several downstream prosurvival and antiapoptotic proteins (c-Myc, Mcl-1, and survivin) were also upregulated at both the mRNA and protein levels. These molecular changes in pulmonary arterial tissues were coincident with pathogenic enhancement of pulmonary vasculature remodeling and elevated pulmonary arterial pressure. Furthermore, flavopiridol significantly alleviated pulmonary artery remodeling and reversed the progression of PH through the inhibition of CDK9-mediated transcription elongation. Taken together, these findings elucidated the potential role and underlying mechanism of CDK9 in the pathogenesis of pulmonary vasculature remodeling under PH, and flavopiridol partially reversed pathogenic vasculature remodeling in PH rats by targeting CDK9.

Growing evidence indicates that PH shares several similar pathogenic characteristics with cancers [6, 7], and one of the most important similarities being that both cancer cells and PH-associated vascular cells, especially PASMCs, exhibit a proproliferative and antiapoptotic phenotype [3, 6]. In the present study, the level of a cell proliferation biomarker (PCNA) in pulmonary arteries from MCT-induced PH rats was increased, and a significant hypertrophic media layer harboring proliferative PASMCs was observed in pulmonary small arteries. This overproliferation phenotype of vascular cells, especially PASMCs, will lead to an increased thickness of the pulmonary artery media wall, consequently promoting PH and right ventricle failure. Moreover, HPASMC overproliferation and resistance to apoptosis were also induced under hypoxic (3%) conditions, similar to that observed in cancer cells exposed to a hypoxic environment [32]. Interestingly, this abnormal overproliferative and antiapoptotic phenotype of PAMSCs was reversed by treatment with the antitumor agent, flavopiridol, in both the in vivo PH model and in vitro HPASMCs, suggesting that reversing these cancer-like phenotypes may be a promising therapeutic strategy to combat PH.

CDK9 is an emerging therapeutic target for cancer treatment, and upregulation of CDK9 has been identified in several cancers [18, 20], such as osteosarcoma, melanoma, and gastric cancer [16, 17, 33]. Consistent with these results, upregulation of CDK9 was also found in our study both in isolated pulmonary arterial tissues from MCT-induced PH rats in vivo and in hypoxic cultured HPASMCs in vitro. Another research group has also reported that CDK9 mRNA expression is increased in both mouse and rat experimental PH models [34], which further supports our hypothesis that CDK9 is also an important therapeutic target for PH. Furthermore, enhanced CDK9-mediated transcription elongation increases the expression of prosurvival and antiapoptotic proteins, such as c-Myc, Mcl-1, and survivin, thereby contributing to the proproliferative and antiapoptotic phenotype of cancer cells [12, 18, 20]. CDK9 inhibitors, such as flavopiridol, SNS-032, and roscovitine, have been demonstrated to be efficient in cancer treatment through downregulation of these prosuvival and antiapoptotic proteins [16, 26, 35]. Similarly, in our study, CDK9-mediated transcription elongation was also enhanced in pulmonary arterial tissues from MCT-induced PH rats and hypoxia-treated HPASMCs, and the enhancement was associated with upregulation of the abovementioned prosurvival and antiapoptotic proteins, indicating that inhibition of CDK9-mediated transcription elongation and decreased expression of these downstream target proteins may also be efficient in the treatment of PH.

Flavopiridol is a broadly specific CDK inhibitor with a distinct preference for CDK9. The *K*_i_ values of flavopiridol for CDK9/CycT (3 nM) have been previously shown to be approximately tenfold lower than those for other CDKs (40–70 nM) [36]. CDK9 inhibition by flavopiridol can remarkably attenuate tumor growth in vitro and in vivo [26, 27]. As previously observed in cancer cells [18], CDK9 upregulation was also detected in both pulmonary arterial tissues from MCT-induced PH rats and hypoxia-treated HPASMCs in the present study. Additionally, CDK9 inhibition by flavopiridol attenuated the overproliferation and promoted the apoptosis of PASMCs, thereby alleviating pulmonary vasculature remodeling and reversing the progression of PH in rats similar to its antitumor effects [18, 20]. Taken together, these results showed that CDK9 upregulation also contributes to the overproliferative and antiapoptotic phenotypes of PH PASMCs similar to that observed in cancer cells.

As a catalytic subunit of P-TEFb, CDK9 activation promotes transcription elongation by phosphorylating RNA polymerase II CTD. In the present study, consistent with its potent inhibition of CDK9 kinase activity observed in cancer cells [26], flavopiridol also significantly reduced the phosphorylation of RNA polymerase II CTD at Ser-2 in isolated PH pulmonary arterial tissues, indicating a potential role of flavopiridol in reversing pulmonary vessel remodeling. Furthermore, the overexpression of several short-lived prosurvival and antiapoptotic proteins, such as c-Myc, Mcl-1, and survivin has been reported to contribute to the overproliferation and resistance to apoptosis phenotypes of cancer cells [19, 20], and these proteins are preferentially depleted by the inhibition of CDK9-mediated transcription elongation [13]. In agreement with the results of other studies [21, 22], both the mRNA and protein levels of c-Myc, Mcl-1, and survivin were also significantly increased in our isolated PH pulmonary arterial tissues. As described in previous studies, downregulation of these prosurvival and antiapoptotic proteins (e.g., survivin) decreases proliferation, induces apoptosis of PASMCs in vitro, and attenuates pulmonary artery hypertrophy, decreases pulmonary vascular resistance, and reverses the progression of PH in vivo [37]. Consistent with these results, in our study, inhibition of CDK9 by flavopiridol also significantly decreased the expression of prosurvival and antiapoptotic proteins as well as induced apoptosis and decreased the proliferation of PASMCs. Therefore, our results suggested that the antiproliferative and proapoptotic effects of flavopiridol on pulmonary arteries and PASMCs are mediated by the inhibition of CDK9-mediated transcription elongation and the expression of the abovementioned downstream target proteins.

In addition to changes in CDK9 expression or activity, CDK9/P-TEFb-related regulators may also be involved in CDK9-related diseases (e.g., cancers) [28, 30, 38]. As previously reported, LARP7, HEXIM1 or HEXIM2 and MePCE, bind to 7SK small nuclear RNA (snRNA) to form the 7SK small nuclear ribonucleoprotein (7SK snRNP) complex, which plays a role in inhibiting CDK9/P-TEFb activity [29, 30]. LARP7 has been previously reported to suppress CDK9/P-TEFb activity, and LARP7 knockdown or inhibition increases CDK9 activity [30]. HEXIM1/2 inhibits CDK9 kinase activity in a 7SK snRNA-dependent manner [29]. MePCE has been reported to stabilize 7SK snRNA to facilitate the inhibition of CDK9/P-TEFb activity [39]. In agreement with these findings, the present study demonstrated that the 7SK snRNP complex components [LARP7, HEXIM1 (not HEXIM2), and MePCE] were significantly decreased in isolated PH pulmonary arteries, suggesting that these CDK9-related negative regulators may also be involved in the pathology of PH. Additionally, previous studies have reported that overexpression of these CDK9-related negative regulators (e.g., LARP7) decreases CDK9/P-TEFb kinase activity to reverse the progression of CDK9-related diseases [30, 38]. Thus, the underlying mechanisms of these CDK9-related negative regulators in PH require further investigation.

Similar to other anticancer agents, however, flavopiridol (0.5 μM) was still cytotoxic toward the control HPASMCs, demonstrating that some therapeutic strategies are needed to reduce the dose of flavopiridol to treat PH. In the present study, three assayed CDK9-related negative regulators (LARP7, HEXIM1, and MePCE) were downregulated in isolated PH pulmonary arterial tissues. Future studies will investigate whether the overexpression of these proteins combined with CDK9 inhibition (flavopiridol) exert synergistic inhibitory effects toward CDK9-mediated transcription elongation to yield a more pronounced suppression of the development of PH and decrease side effects.

There were several limitations of the present study. First, due to the technical simplicity and reproducibility advantages, an MCT-induced PH model was used in the present study, but the differences between MCT models and human PH should be taken into consideration. MCT not only induces PH but also affects both the right and left ventricles as well as other organs (liver and kidney injuries), which may affect the progression of PH [40]. In subsequent studies, analyses of pulmonary artery samples from PH patients may be more convincing to evaluate the associated role of CDK9. Second, flavopiridol is a pan-CDK9 inhibitor and may alter transcription via other mechanisms in addition to CDK9 inhibition or may inhibit other CDKs but with lower efficacy [36]. Thus, it would be more convincing if a more specific CDK9 inhibitor (e.g., BAY-1143572) was used in subsequent studies [41]. Third, P-TEFb comprises CDK9 and cycin T (T1, T2a, or T2b), and cyclin T1 is the primary CDK9 partner cyclin (approximately 80%) [39]. In addition, cyclin T1 has also been reported to be overexpressed in cancer tissues compared to normal tissues, enhancing transcription elongation, and promoting tumor malignances [42]. However, whether cyclin T1 is upregulated and synergizes with CDK9 upregulation to facilitate the development of PH requires further investigation.

In summary, CDK9 upregulation and the subsequent enhanced activation of transcription elongation, which leads to increased downstream prosurvival and antiapoptotic protein expression, may be associated with the mechanisms involved in pulmonary artery remodeling and facilitate the progression of PH. Flavopiridol significantly attenuates pulmonary artery remodeling and reverses the progression of PH by inhibiting CDK9-meditated transcription elongation. These findings may provide novel insights into the pathological mechanism of PH, and CDK9 inhibition, such as flavopiridol treatment, should be considered a potential novel therapeutic strategy for PH.

## Data Availability

The data that support the finding of this study are available from the corresponding author upon reasonable request.
